# Quantum chemical data generation as fill-in for reliability enhancement of machine-learning reaction and retrosynthesis planning

**DOI:** 10.1039/d3dd00006k

**Published:** 2023-03-10

**Authors:** Alessandra Toniato, Jan P. Unsleber, Alain C. Vaucher, Thomas Weymuth, Daniel Probst, Teodoro Laino, Markus Reiher

**Affiliations:** a Laboratory of Physical Chemistry, ETH Zurich Vladimir-Prelog-Weg 2 8093 Zurich Switzerland markus.reiher@phys.chem.ethz.ch; b National Center for Competence in Research-Catalysis (NCCR Catalysis), ETH Zurich Vladimir-Prelog-Weg 1-5/10 8093 Zurich Switzerland; c IBM Research Europe 8803 Rüschlikon Switzerland TEO@zurich.ibm.com; d National Center for Competence in Research-Catalysis (NCCR Catalysis), IBM Research 8803 Rüschlikon Switzerland

## Abstract

Data-driven synthesis planning has seen remarkable successes in recent years by virtue of modern approaches of artificial intelligence that efficiently exploit vast databases with experimental data on chemical reactions. However, this success story is intimately connected to the availability of existing experimental data. It may well occur in retrosynthetic and synthesis design tasks that predictions in individual steps of a reaction cascade are affected by large uncertainties. In such cases, it will, in general, not be easily possible to provide missing data from autonomously conducted experiments on demand. However, first-principles calculations can, in principle, provide missing data to enhance the confidence of an individual prediction or for model retraining. Here, we demonstrate the feasibility of such an ansatz and examine resource requirements for conducting autonomous first-principles calculations on demand.

## Abstract 2

We demonstrate and discuss the feasibility of autonomous first-principles mechanistic explorations for providing quantum chemical data to enhance the confidence of data-driven retrosynthetic and synthesis design based on molecular transformers.

## Graphical abstract

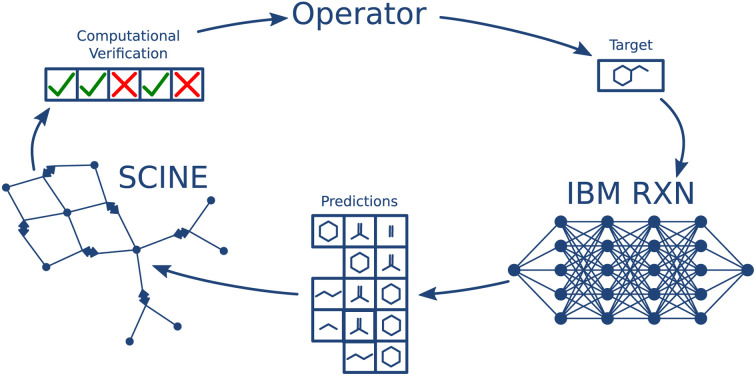

## 1 Introduction

The accurate characterization and reasoning over a diverse range of molecules and chemical reactions is one of the main challenges of artificial intelligence (AI) for chemical research. AI models address a wide range of tasks such as property prediction,^1,2^*de novo* molecular design,^3,4^ and reaction and retrosynthesis prediction.^5–10^ Many of these approaches surpass in accuracy, speed, and scalability the more traditional computational and cheminformatics tools.

The typical strategies of AI-based reaction prediction may be divided into two different groups, namely, template-based and template-free classes. Template-based methods^5,11,12^ rely on a database of reaction templates or rules which are applied to reactant species (or products) to generate transformations. Traditionally, these rules were hand-crafted,^13^ but some AI approaches attempt to automatically extract them from datasets of reactions. By contrast, template-free methods rely solely on the knowledge that can be extracted from raw data and differ in terms of the way they represent the data points: as a graph or as a text sequence.^14,15^ Sequence-based models, in particular, can rely on the relationship between chemistry and linguistics^16–21^ to draw from the large number of publications of AI for natural language processing (NLP). Nam and Kim,^22^ Liu *et al.*,^23^ for example, introduced sequence-to-sequence methods, whereas Schwaller *et al.*^8,9^ proposed transformers for reaction prediction and retrosynthesis. Subsequently, several models leveraging the potential of NLP techniques such as augmentation^24^ and pretraining^25^ have been developed for retrosynthesis or related chemistry tasks.

Irrespective of the approach employed, AI methods suffer from the intrinsic uncertainty of the dataset used for training. The models are usually built with knowledge extracted from publications and patents. Some of the resulting databases of this extraction are the open source USPTO,^26^ the proprietary Reaxys,^27^ and Pistachio.^28^ While these databases provide an extremely valuable set of millions of reactions, they are usually not hand-curated and often imbalanced. This results in models that struggle (or miss) to learn rare and under-represented reactions.

One way to deal with the problem is to validate low-confidence AI predictions by producing reliable data for missing or under-represented regions of the chemical space. This can for instance be achieved with first-principles calculations of the chemical processes in question. Unfortunately, these calculations come at a high computational cost, which even scales the more unfavorable with system size the higher their accuracy shall be. Since, in cases of low-confidence predictions, AI-based models would require such data on demand and with as little time delay as possible, the acceleration and automation of quantum chemical results will be key.

For highly uncertain reaction prediction of an AI model, the automatic quantum chemical (QC) search of chemical reactions with double-ended methods can deliver, first, specific feedback on specific reactions, and second, iteratively enrich the AI training datasets with high-quality data. Within the past decade, approaches have been developed for the automated exploration of reactive chemical systems.^29–35^ A software for this purpose is SCINE Chemoton,^36,37^ which next to fully fledged exploration protocols provides a range of so-called double-sided transition-state search algorithms, that is algorithms which search for a reaction given both reactant and product structures.

SCINE Chemoton is designed as a general-purpose exploration software. Hence, it is not restricted to a particular class of reactions or compounds. The software is under continuous development and is therefore currently affected by some limitations: for instance, the handling of bifurcations along reaction paths is currently not available. The success rate, *i.e.*, the ratio of successfully recovered reactions, was measured at a generic test set to be up to 80% (the missed 20% reactions can be attributed to still lacking reaction types (bifurcations) and deficiencies in the electronic structure model applied in the assessment).^37^ We refer the interested reader to ref. 37 for a more detailed description of Chemoton.

For the sake of completeness, we note that other approaches for the exploration of chemical reaction networks have been developed. Examples are graph-based approaches,^38,39^ relying on chemical heuristics to extract reaction rules^36,40,41^ and stochastic methods^42,43^ (see also ref. 30 and 31 for reviews).

Maeda and coworkers have long highlighted the value of reaction exploration software for retrosynthetic applications.^44–48^ In particular, they presented an approach in which an exploration is started from the desired end product, *i.e.*, the compound which is to be synthesized. Then, an exploration software is taking advantage of automatic identification of possible reaction pathways leading to this compound. We emphasize that the approach we introduce in this work differs with respect to the retrosynthetic analysis that is carried out by an AI prediction to which the quantum chemical validation in the forward direction can be seamlessly coupled in an autonomous fashion.

Another example is autodE^49^ which requires only the SMILES strings of all reactant and product structures. It features a sophisticated approach for finding the transition state and properly takes into account the conformational flexibility of all structures. However, transition state search algorithms are generally only applicable to individual elementary steps, *i.e.*, reactions with only a single transition state and no intermediates. Hence, when some intermediates of a multi-step reaction are not known, such basic approaches cannot be applied. By contrast, explorative approaches such as those implemented in SCINE Chemoton can autonomously discover missing intermediates and, hence, can also be applied to multi-step reactions.

In this work, we explore the feasibility of quantum chemical data production on demand, when requested by artificial intelligence approaches directed to questions of retrosynthesis and synthesis planning. The key goal is to make on-time QC calculations a guide to support decision making of an artificial intelligence-driven model for chemical synthesis planning and mechanism elucidation, not posing constraints on the type of reactions and on their complexity.

The manuscript is structured as follows: Section 2 conceptually introduces our integrated AI-QC framework and discusses the challenges for the implementation and for the interface of the two technologies at play (IBM RXN platform^9,50^ for AI-based retrosynthesis and SCINE Chemoton for double-ended reaction network exploration). In Section 3, we investigate proof-of-concept results for two organic reactions (a Williamson ether synthesis which proves the general feasibility of our approach, and a more complex Friedel–Crafts reaction which is deliberately chosen to push the QC validation to its current limit since the implementation is expected to be very challenging for enriching the AI model with the desired information, thereby highlighting the technical challenges to be addressed by future work on the QM side). We then present thorough resource estimates and discuss the scalability of our framework in a production environment.

## 2 Methodology

### 2.1 Conceptual analysis

Although computational tools for retrosynthesis planning successfully demonstrated how to make arbitrary compounds,^13^ there is an inherent uncertainty regarding the synthetic feasibility of each suggested step. On the other hand, this uncertainty plays a significant role when deciding on a particular synthetic route, as the viability of each proposed reaction step is crucial for overall success. It will especially be important to know the uncertainty associated with the feasibility of reaction steps that turn out to be pivotal or even affect important resource requirements in earlier steps of a chemical synthesis. Monitoring the feasibility of each step is therefore important and requires automated uncertainty assignment.

One possible way to validate and prioritize reactions is to rely on confidence measures provided by AI-based retrosynthesis models. This value is usually obtained by a complementary forward reaction prediction model and is therefore indicative of the probability that a suggested reaction will work.^9^ However, the resulting confidence largely depends on the data on which such data-driven models were trained. Information linked to under-represented and noisy data instances can be more difficult to learn,^51^ which results in models assigning a low confidence to the corresponding reactions. In such a case, one must resort to additional data sources to gain more insight into these under-represented data points. However, experimental data on specific reactions will, in general, not be readily available.

**Fig. 1:**
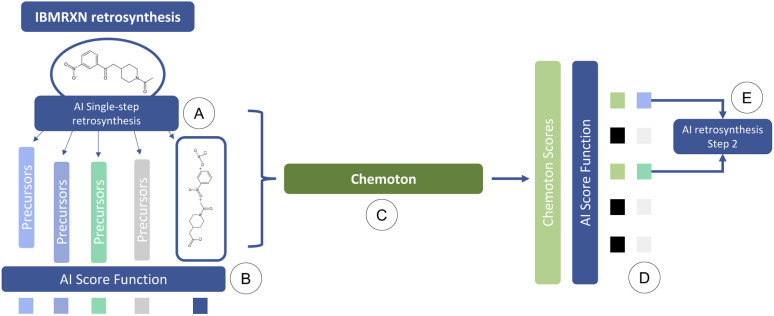
Workflow of QC-enhanced AI-based retrosynthesis planning: (A) a prediction of possible disconnections is made for a target molecule. (B) A confidence score is computed for these predictions. Some of these suggestions are potentially correct but predicted with low confidence by the AI model (in IBM RXN) due to a lack of training data. (C) First-principles reactivity explorations (with SCINE Chemoton) are initiated to validate or invalidate the predictions (or a subset of them). (D) The original confidence score and the result of the first-principles explorations are combined to decide which predictions should be adopted for the synthesis planning. (E) The above procedure is iterated for the next steps.

To address this issue, we propose the following integrated AI-QC framework, where we leverage first-principles calculations. Through reaction explorations relying on the SCINE Chemoton^37,52^ platform, we set out to fill the gap of missing data and prediction uncertainty in AI-based retrosynthesis models, using as an example, those based on language architectures.^9,50^ When a retrosynthetic route provided by the AI model is deemed to be unsatisfactory in terms of confidence, a new retrosynthesis search is performed, where reaction network searches are triggered in the background on each single-step prediction. The results of the reaction network searches are then inspected to validate or invalidate the suggested retrosynthetic suggestions and update the corresponding confidences. These updated values are integrated into the multi-step retrosynthesis algorithm and the details of the reactivity exploration are provided to the users, who can then proceed to the next steps in the synthesis. This AI-QC framework is sketched in Fig. 1, where for the sake of convenience only a single reaction step is reported.

We emphasize that quantum chemical reaction network explorations can be computationally very demanding, potentially becoming a critical bottleneck of our approach. We therefore discuss resource estimates for reaction network searches and their feasibility for routine applications in Section 3.3.

### 2.2 Implementation

For demonstrating the integration of data-driven retrosynthesis and first-principles network exploration, we rely on the IBM RXN retrosynthesis algorithm^9,50^ and on SCINE Chemoton.^37,52^ Since both technologies have been presented in the original publications cited above, we refer the interested reader to Appendix A for a short description of both. Instead, in this section, we will look at how these technologies can be combined and the challenges that will be encountered. In particular, we discuss what information should be provided for the first-principles validation of a reaction suggested by the data-driven retrosynthesis tool and how this information can be provided on demand.

To start a first-principles network exploration, SCINE Chemoton will require some minimal specification (minimal input). However, the minimal input will spur many explorative calculations that can be easily in conflict with demands on speediness and hardware resources. Therefore, Chemoton also accepts additional parameters that lead to a more directed search and reduce the number of necessary calculations, hence making the network exploration faster.

The minimum input needed by SCINE Chemoton to start an exploration consists of the Cartesian coordinates of structures for all compounds in the reaction, including solvent and catalysts, as well as the total molecular charge and spin multiplicity for all of these structures. This information is indirectly provided by the retrosynthesis tool, which suggests the precursors in the simplified molecular-input line-entry system (SMILES) notation.^53,54^ The SMILES format specifies the connectivity of the compounds, but not their three-dimensional coordinates. The conversion to three-dimensional coordinates can follow existing approaches.^55–57^ In some cases, special care is needed to avoid individual atoms overlapping; such structures, if not properly sanitized, lead to failing quantum chemical calculations. The total molecular charges are obtained directly from the SMILES strings, while the spin multiplicity can be guessed automatically by some very fast (but crude) methods, for example, one based on extended Hückel theory.^58^ In a more sophisticated approach, all possible low-lying spin states need to be tested as has already been proposed for molecular propensity calculations.^59^

Moreover, one should be aware of the fact that a retrosynthesis tool may fail to provide the stereochemical information for a molecule. In some cases, this information is simply not available (*e.g.*, because it was already lacking in the input data). In other cases, certain stereochemical information, such as the axial chirality of a helicene molecule, cannot be encoded in SMILES notation.^60^ A straightforward way to deal with missing stereochemical information from the AI model is to account for all options in the first-principles exploration; *i.e.*, to use all possible stereoisomers in separate Chemoton explorations.

While the single-step retrosynthesis models were designed to predict catalysts and reagents in addition to the reactants,^9^ there is no guarantee that the data used to train the model (or that the model predictions) are complete. Then, help may be provided by additional data-driven models, such as models providing the missing compounds in incomplete chemical equations.^61^

Since SCINE Chemoton cannot steer an exploration into a desired direction based on the minimal input, the combinatorial explosion resulting from attempts to react every atom and atom pair in every molecule with every atom and atom pair in every other molecule will be computationally demanding and must be tamed. By providing Chemoton with additional information on a reactive system under consideration, the exploration can be made much more efficient.

First, the atom mapping between reactants and products, *i.e*., the set of rules defining how atoms rearrange during a chemical transformation, can reduce the number of atom combinations that Chemoton needs to explore, in particular when the reaction is composed of a single step (if the reaction turns out to be composed of multiple intermediate steps, an extensive network exploration should be performed). There exist many computational tools for atom-to-atom mapping.^62^ One of them, RXNMapper,^63^ relies on a similar architecture as the single-step retrosynthesis model and can easily be queried to enhance the suggested set of precursors with atom mapping information. Here, “precursors” refer to the set of reactants and reagents proposed for a single-step synthesis from the AI retrosynthesis tool. Second, from the list of precursors, the roles of the individual structures (*e.g.*, reactant, solvent, catalyst) are not known. Knowing this information can improve the first-principles network exploration because one would be able to model solvent effects adequately or rely on the fact that a catalytic species is to be recovered at the end of the reaction. We foresee that data-driven models, for instance following an approach similar to named-entity recognition,^64^ will be able to determine the precursor roles in the future.

Third, one can expand on the previous point to also specify the stoichiometry of the precursors. This is especially relevant in cases where multiple identical reagent molecules are consumed by the reaction. If the stoichiometries are not available, one can allow for free exploration with different stoichiometries of the different precursors. This could, however, lead to an exceedingly large number of calculations. To our knowledge, no computational tool currently exists to estimate the stoichiometry of precursors. We expect this task to be addressable by data-driven models, should adequate training data be available.

Fourth, the knowledge of byproducts, such as condensation water, can be very helpful. If all products of a reaction are known, this can be used to limit the scope of the exploration and, hence, will reduce the computational resources needed to verify whether a given reaction is viable. With state-of-the-art AI models, however, it is hard to determine byproducts automatically. In principle, the aforementioned chemical equation completion^61^ is able to provide missing byproducts. In practice, however, data sets of chemical reactions do not provide this information, which is either unknown or of no practical interest to chemists.

Finally, work-up steps can be relevant when aiming to recover the reaction product specified in the chemical equation. In many reactions, this product will only be recovered after work-up. For instance, for a Grignard reaction, it will not be possible to obtain the final alcohol unless one considers the presence of water after the actual reaction. Since the reagents used for work-up are not always specified in training data and are therefore not available from the AI model, this can prevent Chemoton to recover the reaction product. Strategies to account for such a failure, for instance by relying on data-driven models that are able to predict work-up steps,^65^ need to be studied in future work.

## 3 Results

To assess the feasibility of our AI-QC framework and its integration of data-driven retrosynthetic suggestions and the first-principles validation of their reactions, we studied two examples of different complexity. We start with a single-step example in the next section before we turn to the more complex multi-step problem.

### 3.1 Proof of concept demonstration

As a proof of concept, the first example is deliberately chosen to be a reaction which occurs in a single elementary step. Consequently, one may expect the validation of the reaction by first-principles mechanism exploration to confirm the feasibility of this reaction in comparatively little time. Hence, we also measure the time and resources required for the exploration.

**Fig. 2:**
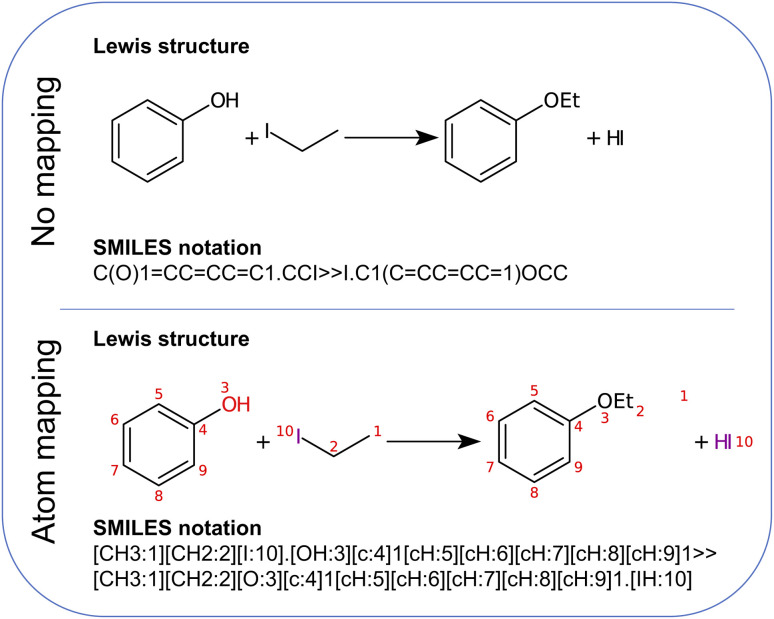
Williamson ether synthesis of ethoxybenzene from iodoethane and phenol. Both the non-mapped and the atom-mapped^63^ Lewis structures are shown alongside the corresponding SMILES representations.

As a target compound, we selected ethoxybenzene (*i.e.*, ethyl phenyl ether). The preferred way to synthesize this compound is a Williamson ether synthesis starting from iodoethane and phenol (see Fig. 2). The mechanism of this reaction is known to be a nucleophilic substitution (S_N_2), occurring in a single elementary step.^66^

The single-step AI-based retrosynthesis model of IBM RXN suggests multiple variants of this reaction with different bases (potassium carbonate and sodium hydride) and solvents (acetonitrile, acetone, and dimethyl formamide). It also suggests a few alternative reactions starting from bromoethane instead of iodoethane. To keep things as simple as possible, we started from a simplified version of this reaction insofar as neither a base nor a solvent is explicitly considered in the exploration with Chemoton. Obviously, in some cases, it might be crucial to incorporate explicitly solvent effects into the exploration. We have recently presented an approach on how this can be achieved,^67^ which can be directly exploited in our context, although further work will be needed to avoid the computational effort of solvating every structure in a network. This simplified version is also suggested by the retrosynthesis tool, although with slightly lower confidence than the alternatives including a solvent and a base.

Since this is a single-step reaction, Chemoton can take advantage of the atom mapping provided by RXNMapper to set up only the minimal number of calculations necessary to probe whether the AI-predicted simplified solvent and base-free reaction is feasible. For instance, in this particular example, it is immediately clear from the atom mapping that it is a bimolecular reaction; hence, Chemoton will not attempt to find any unimolecular reactions. Also, the atom mapping clearly shows that the phenyl moiety is not taking part in the reaction. Hence, this can be excluded as well in the elementary reaction step searches set up by Chemoton. Note that for this proof-of-concept work, the atom mapping was implemented manually. It will be left to future work to automate this.

Chemoton then carried out a total of 729 calculations requiring between 0.03 s and 49.61 s with an average runtime of 9.80 s. The total runtime required for a serial execution (*i.e.*, using a single processor core only) was 7146.13 s or slightly less than two hours. A massive speedup could be obtained by employing several processors in parallel. Upon completion of all calculations, the exploration yielded a total of 43 compounds and 38 reactions (according to the notation in ref. 32, where structures with the same connectivity represent a compound and a reaction will, in general, consist of several alternative elementary steps). The set-up and running of these calculations was done in a fully automated fashion by SCINE Chemoton with no manual intervention.

The first reaction found by Chemoton is the desired target reaction, *i.e.*, the nucleophilic substitution yielding the ester. With the GFN2-xTB method,^68,69^ the reaction energy (as calculated from total electronic energies, hence not taking into account any nuclear motion and entropic effects) is −34 kJ mol^−1^, and the barrier to overcome by the reactants is predicted to be 218 kJ mol^−1^. Note that this barrier is arguably rather large; it results from the fact that no base was present and, hence, the nucleophile was phenol instead of phenolate. This was confirmed by additional calculations carried out with TURBOMOLE 7.4.1 (ref. 70 and 71) in its shared-memory parallelized version in the framework of DFT with the PBE exchange–correlation functional^72,73^ and Ahlrichs' def2-SVP basis set^74^ for all atoms (in addition, semi-classical D3 dispersion corrections^75^ with the Becke–Johnson damping function^76^ were employed). When calculating the same reaction with phenolate instead of phenol, the barrier is lowered to 158 kJ mol^−1^. By contrast, relying on a more sophisticated electronic structure method (density functional theory with the PBE functional and Grimme's D3BJ dispersion corrections with the def2-SVP basis set) while keeping the phenol reactant, the barrier is only slightly reduced (namely, to 203 kJ mol; for phenolate, the barrier remains at 158 kJ mol^−1^ even with DFT as for GFN2-xTB).

Most other reactions are endothermic. These are 34 out of 38 reactions and they can already be ruled out as promising candidate reactions, assuming that the reaction outcome is thermodynamically controlled. The two remaining reactions are more exothermic than the target reaction, namely −48 kJ mol^−1^ and −52 kJ mol^−1^. These reactions are also substitution reactions, but with the difference that the ethyl moiety attaches to the phenyl ring in ortho and para positions to the hydroxy group. However, these two reactions have barriers which are significantly larger than the 218 kJ mol^−1^ of the target reaction, namely 250 kJ mol^−1^ and 253 kJ mol^−1^. Using a Maxwell–Boltzmann distribution to model the kinetic energy distribution of the reactants at 100 °C, we find that the number of molecules having a kinetic energy between 218 kJ mol^−1^ and 250 kJ mol^−1^, *i.e.*, enough to surpass the barrier of the target reaction, but not enough to overcome the ones from the other reactions, is four orders of magnitude larger than the number of molecules having a kinetic energy of more than 250 kJ mol^−1^. Therefore, we conclude that the target reaction is indeed the dominating one, as suggested by the AI-based model in IBM RXN.

### 3.2 A challenging example put to the test

The first example reaction demonstrates the feasibility of our approach. However, for more complex scenarios, there are many technical challenges which need to be solved. We highlight these challenges in the second example below, which is deliberately chosen to push the current implementation of our approach to its limits in order to draw conclusions for future research directions.

As a second, more challenging example, we selected a case that is more difficult both for the AI-based retrosynthesis model and for the first-principles network exploration. It has been shown that the family of Friedel–Crafts acylation reactions is challenging for text-based models for chemical reactivity.^77^

**Fig. 3:**
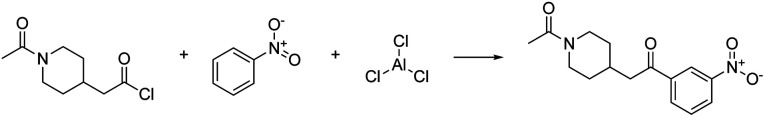
Friedel–Crafts acylation reaction.

The specific reaction we studied is shown in Fig. 3. This is one of the examples considered by Kovács *et al.*,^77^ who observed that the Molecular Transformer,^8^ a forward reaction prediction model, struggled to predict the correct regioisomer depending on the directing group attached to the aromatic ring. Since the Molecular Transformer shares the architecture of the single-step retrosynthesis model and is trained on the same data, this example is expected to be challenging for retrosynthesis as well.

Moreover, while feasible in principle, the above reaction presents other obstacles than those linked to regioselectivity. According to standard organic chemistry textbook knowledge,^66^ one might argue that this reaction is not expected to occur, given that neither the electrophile nor the aromatic moiety (deactivated by the electron-withdrawing nitro group) is particularly reactive. Still, strong heating/microwave may allow for at least some conversion. These considerations make the presented reaction a perfect case study for our analysis.

**Fig. 4:**
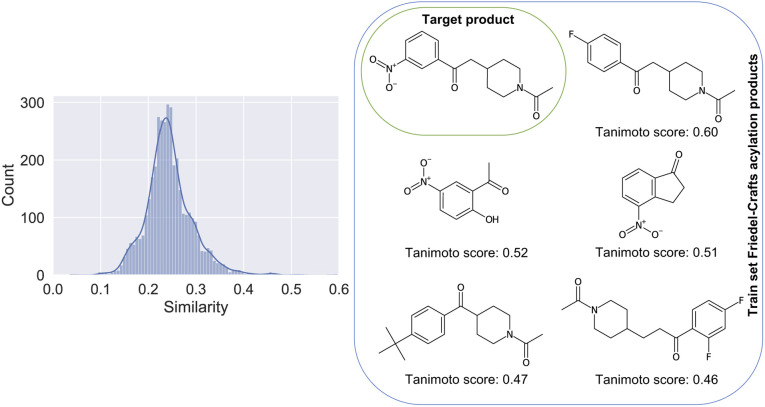
Left: Similarity of all products present in the training dataset and linked to a Friedel–Crafts acylation reaction, plotted against the product of the considered Friedel–Crafts reaction of Fig. 3. Right: The 5 molecules with the highest similarity score against the target compound (top left of the grid). Note that the product with the highest similarity is the one most confusing (F-group in para position *vs.* meta).

The difficulties linked to this example can be rationalized by the poor characterization of these reactions in the patent dataset. There, for training, only 0.21% belong to the class of Friedel–Crafts acylation reactions. In Fig. 4, we show the distribution of Tanimoto scores^78^ of all the product molecules present in the dataset and derived from Friedel–Crafts reactions against the target molecule, including some examples and associated scores. For reactions like this one, new data points generated by on-demand quantum chemical explorations have the potential to provide key information lacking in the dataset.

This example highlights the challenges of an automated verification with Chemoton and several of the challenges mentioned in Section 2.2 apply here. For instance, no solvent is specified, and also no stoichiometries are available. Also, the atom mapping information is of little relevance in this case, because the overall reaction as depicted in Fig. 3 is not a single elementary step.

If one were to rely on the atom mapping to steer the exploration with Chemoton, no reactions would be found, and Chemoton would erroneously report that this chemical transformation was not possible. This result can be prevented if it is known that the overall reaction is composed of several elementary steps. However, as long as the intermediate structures are not precisely known, it is difficult to steer the exploration and limit the computational resources needed. For example, with the default settings of Chemoton 2.1.0, a brute-force exploration starting from the three reactants shown in Fig. 3 quickly results in more than 10 000 calculations. When the scope of the exploration is further broadened by allowing up to two bond modifications, three rotamers, and multiple attack points for every reactive elementary-step trial, the exploration quickly exceeds 1 000 000 calculations.

For such multistep reactions, new methods to identify the individual elementary steps will have to be developed to maintain the exploration within tight bounds and, hence, within reasonable computing time. The development of such ways, however, is beyond the scope of this article and left for future work.

**Fig. 5:**
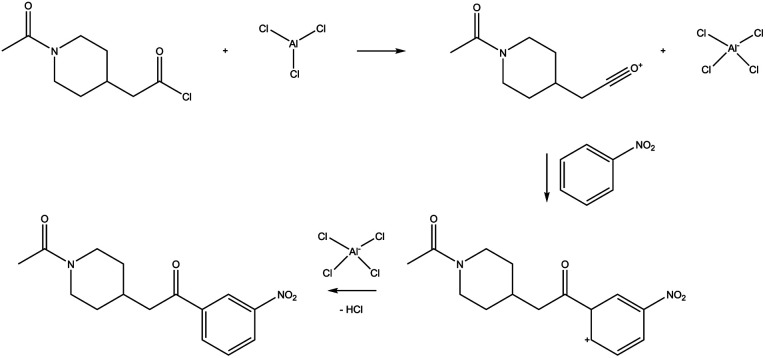
Elementary steps of the Friedel–Crafts reaction shown in Fig. 3.

We explored this reaction manually, breaking it up into the well-known elementary steps of Friedel–Crafts acylation as shown in Fig. 5. Having these elementary steps defined, deducing a corresponding atom mapping, and taking advantage of this in exploration is trivial. However, while the first elementary step was successfully found with Chemoton, repeated attempts to find the second step failed. The failed attempts can be traced back to the scarce reactivity of the species involved, as explained before. However, there is a not small possibility that this reaction could not be found for technical reasons because the fast, but approximate semiempirical electronic structure method employed may fail for this particular example. While more sophisticated methods are readily available in many cases, these require longer computational times.

As a side note, for this specific reaction, the catalyst (AlCl_3_) is typically found in dimeric form (Al_2_Cl_6_), but this is usually not specified explicitly in databases and then cannot be given by the AI-based model to the Chemoton exploration in an automated fashion. If the dimeric form is important for the reaction to proceed, it will be impossible to find in a first-principles exploration unless this dimeric form is given as input, or allowed to create it. The latter option is easy to implement, but will again increase the time needed for the exploration.

### 3.3 Resource estimates and feasibility in routine applications

After having studied a straightforward and complex reaction, we now take a bird's eye position to discuss the general requirements for our AI-QC framework.

For a routine integration of data-driven retrosynthetic models with first-principles network exploration, it is key that the results from the first-principles exploration are provided in a timely fashion to be useful for further decision making with respect to the target molecule. In this respect, it is crucial to understand what results can be expected to be provided by the first-principles exploration in a practical amount of time.

As an example, one can consider a duration of two weeks to be reasonable to obtain a thorough analysis of potential synthesis paths for a molecule of interest. Given that a multistep data-driven retrosynthetic analysis takes a few minutes on average, this will not be a bottleneck, and one can concentrate on the time required for the first-principles exploration. Assuming that two iterations of Chemoton explorations will be necessary, one can assume that the results of each iteration must be provided within one week. On the basis of the observations made in Sections 3.1 and 3.2, this leads to the following considerations.

There are two major factors that will determine the overall computational effort for these explorations. The first is the electronic structure method chosen to evaluate energies and gradients in the explorations. The second is the number of elementary-step search trials that are performed. While the choice of the electronic structure method determines the cost of each energy evaluation in relation to the size of the given molecules, it is nonetheless the number of trials that are ultimately the bottleneck for larger explorations. The number of trials can grow exponentially with the size and number of relevant systems to probe unless these trials are filtered.

Expanding on the runtimes reported in Section 3.1, we can divide the runtimes per calculation into those calculations that are elementary-step search trials and those that are not, the latter usually featuring a much lower runtime than the elementary-step trials. As mentioned above, the cost of each elementary step trial is of key interest for the calculation of the overall cost. The average runtime per elementary step trial in the example case presented in the previous sections is 20.1 seconds. As these timings are obtained on a single core, we can therefore round the cost per reaction trial to 20 CPU seconds or 1/180 CPU hours. This runtime average is calculated from all trials, those that lead to the discovery of a new elementary step and those that did not. The latter are usually of a significantly shorter runtime, as they end the procedure prematurely upon detection of an error or determination of a dead end. We observed that the ratio of successful and unsuccessful trials is almost constant for a chosen set of exploration algorithms,^37^ so we can leverage this average value to extrapolate costs for cases with more trials and similar system sizes.

Coming back to the intended constraint of finishing an exploration within a week, it would therefore be possible to probe 30 240 elementary step trials on a single core in this time frame. If more computational power is invested within the same time, this number may of course be increased. Assuming this task should be a rather routine job, we may set the limit that no more than 100 cores should be invested, hence generating an upper limit at about 3 million reaction trials within one week. Compared with the examples given above, these calculated trial numbers would make it appear that it is possible to tackle many problems, but this estimate was generated for the given system size and method used in those examples.

Should the exploration rely on pure DFT calculations for these examples, we would encounter clear limitations. Depending on the system size a slowdown of a factor of 100 to 1000 can be expected, hence allowing only very limited cases to be tackled within the one-week limit. This is, of course, a problem for chemical processes that are not well described with current semi-empirical methods.

Furthermore, with increasing system sizes, the computational cost of each trial also rises. Assuming a quadratic scaling of the energy and gradient evaluations for the semi-empirical methods with respect to system size shows that doubling the system size may yet be feasible from the point of view of electronic structure methods. However, increasing system sizes also increases the number of trials required to cover all possible reactions for the given systems. Even if we only assume the possibility of forming a single bond in each bimolecular trial, this scaling will be at least quadratic in the size of the systems involved, as every atom of one reactant could potentially form a bond with every atom of the other reactant. Overall, the doubling of the system sizes will therefore cut the coverage of reactions within a single week by a factor of 16.

A need for further steps is obvious to tame the combinatorial explosion of search trials. Some of this may be based on additional information generated by the initial AI-based step. Other parts may be based on first-principles heuristics.^41,79,80^ Combined with the ongoing developments in faster and/or more accurate electronic structure methods, in recent years also including machine learning approaches for energies and gradients, we expect that these computational cost-based problems will be alleviated in the future.

## 4 Conclusions

Deep learning approaches to retrosynthesis prediction have long been travelling on separate routes with respect to more traditional quantum chemistry approaches like reaction network searches. In our work, we aimed at finally merging the two technologies, highlighting how both worlds can benefit from one another. We have presented a new framework where first-principles calculations provide missing data to enhance the confidence of an individual prediction or for model retraining. We have examined the bottlenecks at interfacing a SMILES-based output to a more complex (minimal) input like the one required by first-principles calculations, underlying the importance of the generation of three-dimensional conformer structures, the presence and identification of certain species in the reactions (*e.g.*, solvent, catalyst, by-products) and the knowledge of eventual intermediate steps. Through two prototypical examples (Williamson ether synthesis and Friedel–Crafts acylation), we demonstrated the feasibility of such symbiotic technology and estimated truthfully resource requirements for a routine integration of data-driven retrosynthetic models with first-principles network exploration. We envision a hybrid platform where the user can define her/his degree of granularity in the synthesis of a target molecule, leveraging the broad knowledge and speed of AI models as well as the accurate and thorough analysis of first principles calculations.

## Appendix

### A Methods

#### A.1 Data-driven retrosynthetic route prediction

Modern AI-based approaches to multistep retrosynthesis typically rely on a Deep Learning single-step retrosynthesis model, coupled with a search algorithm. In this work, we followed the approach by Schwaller *et al.*,^9^ as implemented on the IBM RXN platform.

The single-step retrosynthesis model is a language model constructed with the popular Transformer architecture by Vaswani *et al.*^81^. Molecules and reactions are represented as SMILES strings and fed to the model token-by-token (tokenization is performed by splitting the SMILES characters, see Schwaller *et al.*^9^ for more information). The model was trained on a proprietary patent dataset, Pistachio,^28^ and it learned to generalize to unseen molecules and reactions.

**Fig. 6:**
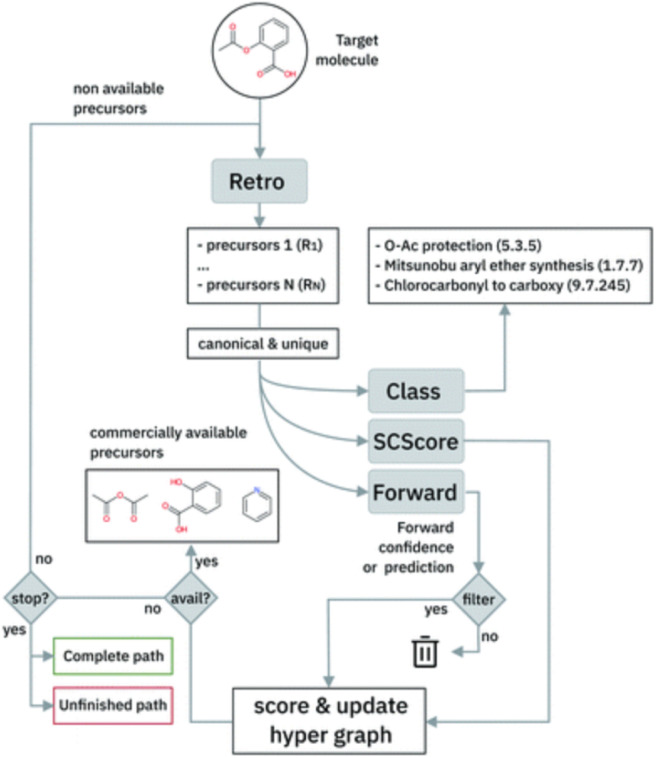
Multistep retrosynthesis algorithm logic, taken from Schwaller *et al.*^9^

The single-step model is embedded in a Beam Search algorithm to perform the path exploration. The algorithm is briefly sketched in Fig. 6. The single-step retrosynthesis model suggests a series of possible disconnections for a target molecule. The proposed sets of reactants are scored in terms of a scoring function based on the properties of the chemical species (SCScore^82^) and on the confidence of the model on that prediction (‘Forward’ in Fig. 6). The Beam Search algorithm iterates the procedure for multiple subsequent steps (until available precursors are found or a maximum number of steps is reached).

**Fig. 7:**
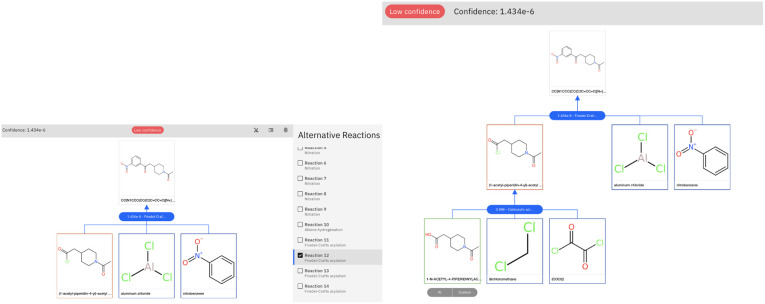
Graphical user interface view for the result of the retrosynthetic analysis of the product molecule of Section 3.2. Left: selection of the first reaction step, which is characterized by a low confidence. Right: global view for the full retrosynthetic route. The low confidence score is caused by the first reaction step.

The retrosynthetic tool is available for public use through the IBM RXN GUI.^50^ To run the examples in this paper, we used the model categorized as ‘2020-07-01’ and run the multistep synthesis in interactive mode. We report in Fig. 7 the graphical-user interface output for the challenging example of Section 3.2.

The low confidence of the first step (Friedel–Crafts acylation) is highlighted in the user interface. The prompt intervention and validation by first-principles calculation can prioritize this reaction and allow it to proceed smoothly with the carboxylic acid to acid chloride reaction, proposed by AI and leading to commercial precursors.

#### A.2 Automated first-principles reaction mechanism exploration

All quantum chemical explorations were carried out with Chemoton 2.1.0 (ref. 37 and 52) in conjunction with Puffin 1.1.0.^83^ Reactive trials were generated with the NT2 algorithm.^37^ Only bimolecular trials were created, allowing one intermolecular bond formation, and excluding any intramolecular bond formations and any dissociations. All atoms except hydrogen were considered reactive. For every reactive trial, two rotamer structures and multiple attack points were employed.

The quantum chemical raw data (*e.g.*, electronic energies and nuclear gradients) were obtained from GFN2-xTB.^68,69^ All calculations were done in the restricted open-shell formalism and the C_1_ point group symmetry.

## Data availability statement

The data underlying the results presented in this article is available on Zenodo.^84^

This exploration has been carried out with Chemoton 2.1.0 in conjunction with Puffin 1.1.0. Note that this implies the following versions of the entire SCINE Chemoton exploration stack:

– Chemoton 2.1.0 ^85^

– Puffin 1.1.0 ^86^

– Database 1.1.0 ^87^

– ReaDuct 4.1.0 ^88^

– Molassembler 1.2.1 ^89^

– Utilities 6.0.0 ^90^

– Core 4.0.2 ^91^

– development-utils 5.0.1 ^92^

– xtb_wrapper 1.0.2 ^93^

All quantum chemical raw data needed for this exploration (such as electronic energies and nuclear gradients) have been obtained with the xtb program.^94^

The additional verification calculations for the transition state in Sec. 3.1 have been carried out with ReaDuct 4.1.0 ref. 88 in conjunction with xtb^94^ for the GFN2-xTB^95^ calculations and with Turbomole 7.4.1 ^96,97^ for the DFT calculations (in a def2-SVP basis set^98^ at all atoms and with the PBE0 exchange–correlation density functional^99^). Dispersion effects were considered by Grimme's semiclassical D3 correction^100^ with Becke–Johnson damping function.^101^

The retrosynthesis predictions were obtained from the freely available IBM RXN for Chemistry platform.^102^ The underlying AI models were presented elsewhere^103^ and rely on the transformer model implementation available in the OpenNMT-py package.^104^ A reference implementation for the application to chemical reactions is provided by the Molecular Transformer.^105^

The retrosynthesis model was trained on patent data obtained from Pistachio, a proprietary database by NextMove Software.^106^

## Conflicts of interest

There are no conflicts to declare.

## Supplementary Material
